# Deoxynivalenol induces structural alterations in epidermoid carcinoma cells A431 and impairs the response to biomechanical stimulation

**DOI:** 10.1038/s41598-018-29728-5

**Published:** 2018-07-27

**Authors:** Giorgia Del Favero, Lydia Woelflingseder, Lukas Janker, Benjamin Neuditschko, Stefano Seriani, Paolo Gallina, Orfeo Sbaizero, Christopher Gerner, Doris Marko

**Affiliations:** 1 Department of Food Chemistry and Toxicology, Faculty of Chemistry, University of Vienna, Währingerstr. 38-40, 1090 Vienna, Austria; 2 Department of Analytical Chemistry, Faculty of Chemistry, University of Vienna, Währingerstr. 38-40, 1090 Vienna, Austria; 3 Department of Engineering and Architecture, University of Trieste Via A, Valerio 10, 34127 Trieste, Italy; 4 Robotik und Mechatronik Zentrum, Deutsches Zentrum für Luft- und Raumfahrt e.V. (DLR), Oberpfaffenhofen, Germany

## Abstract

Morphology together with the capability to respond to surrounding stimuli are key elements governing the spatial interaction of living cells with the environment. In this respect, biomechanical stimulation can trigger significant physiological cascades that can potentially modulate toxicity. Deoxynivalenol (DON, vomitoxin) is one of the most prevalent mycotoxins produced by *Fusarium* spp. and it was used to explore the delicate interaction between biomechanical stimulation and cytotoxicity in A431 cells. In fact, in addition of being a food contaminant, DON is a relevant toxin for several organ systems. The combination between biomechanical stimulation and the mycotoxin revealed how DON can impair crucial functions affecting cellular morphology, tubulin and lysosomes at concentrations even below those known to be cytotoxic in routine toxicity studies. Sub-toxic concentrations of DON (0.1–1 μM) impaired the capability of A431 cells to respond to a biomechanical stimulation that normally sustains trophic effects in these cells. Moreover, the effects of DON (0.1–10 μM) were partially modulated by the application of uniaxial stretching (0.5 Hz, 24 h, 15% deformation). Ultimately, proteomic analysis revealed the potential of DON to alter several proteins necessary for cell adhesion and cytoskeletal modulation suggesting a molecular link between biomechanics and the cytotoxic potential of the mycotoxin.

## Introduction

The integration of biomechanical stimulation in cytotoxicity testing is an approach that is becoming more and more frequent^1–4^. In fact, under physiological conditions, cells are continuously exposed to mechanical stimulation, as a result of both, the movement of the tissues/organism to which they belong and the flow of the extracellular fluids. In this respect, mechanical stimulation can actively modulate cellular physiology, but the impact of these effects in the response to xenobiotics is rarely taken into consideration. Indeed, the knowledge about the impact of biomechanical stimulation on the toxicity is limited in comparison to the numerous *in vitro* studies performed in static conditions. In general terms, if the potential impact of stretching in cells like myocytes^5–8^, or vascular endothelial cells^9–13^ is very easy to foresee, more and more studies describe that also other cell types can modulate their responses, if cultured in a mechanically stimulated environment^14–16^.

Deoxynivalenol (DON) is one of the most common food contaminating mycotoxins^17–19^. It is regularly detected in food commodities all over the world^20–24^ and has been already associated to several cases of intoxication^25^. DON is known to act primarily on protein synthesis, blocking the ribosomal subunit 60S^26^. Obviously, the inhibition of protein synthesis can have impact on a wide variety of cellular physiological processes, and the biological effects of DON have been extensively studied in numerous *in vitro* models^26–29^, but typically in static conditions. In this respect, many cytotoxicity assays are routinely performed measuring several endpoints like protein content, membrane permeability and functionality of cellular organelles^30,31^. In more detail, cytotoxicity studies commonly evaluate mitochondria or lysosome function alone or in combination^31–33^. Lysosomes are acidic organelles that play a crucial role in the turnover of cellular components and autophagic degradation^34,35^. Proper cellular function of these organelles is tightly related to their spatial localization and their interaction with tubulin microtubules^36–39^. Recent studies connected the importance of autophagic processes to biomechanical responses^40,41^. Similarly, the role of autophagy in the toxicity of DON has recently been highlighted^42,43^, but the interplay of these processes in a more complex environment, thus characterized by mechanical stimulation has never been addressed. Therefore, in the present study the potential connection between the effect of the trichothecene mycotoxin DON and the alteration of the lysosome function is investigated through the interaction with the cytoskeleton. Moreover, the interplay between the biomechanical stimulation and the cytotoxicity of DON is explored comparing directly static incubations and cyclic uniaxial stretching. To this aim, we used a prototype device^44^, designed and built for the application of cyclic uniaxial stretching to cultivated cells. In fact, biomechanical stimulation is known to have a positive impact on the cellular cytoskeleton and trophic stimuli sustaining, among others, cellular proliferation and survival^5,45^. In the present study the effect of DON was investigated on the epidermoid carcinoma A431 cells, since, in recent times, DON has been described also for its effects at dermal level^46,47^ and A431 cells have been extensively used as a model for studies on the effect of natural compounds in human skin cell models^48,49^. A431 cells are characterized by excellent adhesion properties which are crucial for the consistent performance of experiments in mechanically stimulated environment. Particular attention was given to the evaluation of the functional properties of A431 cells after exposure to different concentrations of DON, establishing a workflow based on the evaluation of living cells. Moreover, the study was completed by the evaluation of the effect of DON on the proteome profile of A431 cells. The capability of the mycotoxin to impair the biomechanical properties of A431 cells is described for the first time, opening a new horizon in the importance of the integration of biomechanical stimulation in toxicity testing also in the field of mycotoxins.

## Results

### Toxicity of DON in A341 cells: impact on tubulin cytoskeleton

**Figure 1:**
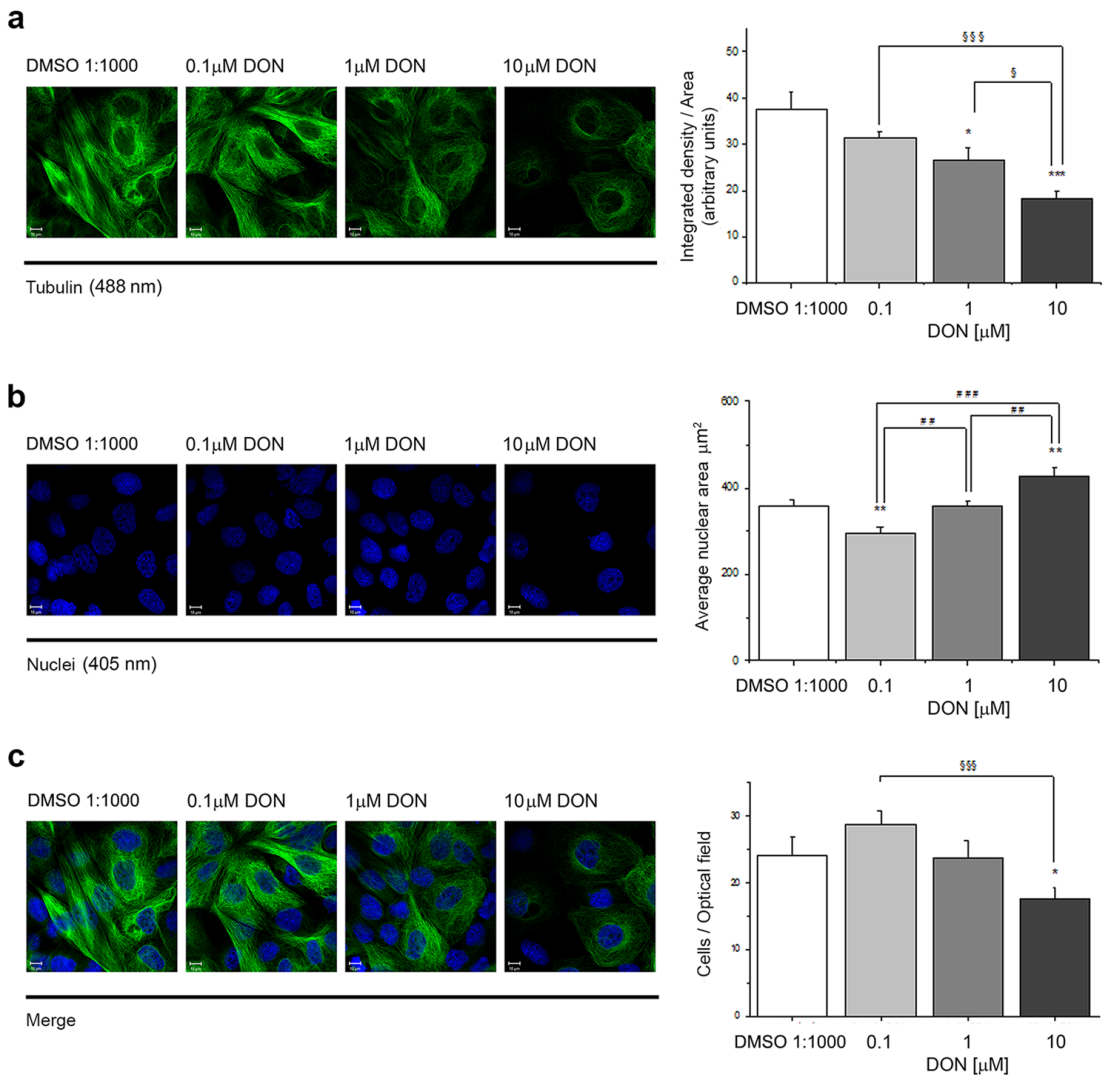
Effect of DON on the tubulin cytoskeleton of A431 cells. (**a**) Representative images of immunolocalization of tubulin (green) and relative quantification of the mean integrated density of the fluorescence signal/area. (**b**) Cell nuclei counterstained with DAPI and nuclear area quantification. (**c**) Merged images and quantification of cells/optical field. Scale bars are equivalent to 10 µm. *Expresses significant difference in comparison to controls (white bars – DMSO 1:1000; *p < 0.05, **p < 0.01, ***p < 0.001; ^§^expresses significant difference in comparison to respective concentrations of DON (grey bars; ^§^p < 0.05, ^§§^p < 0.01, ^§§§^p < 0.001). Data are expressed as mean ± S.E. of n = 4 independent experiments analyzing for every experiment minimum 3 different optical fields.

The cytoskeleton is a key element governing cellular biomechanical properties, thus preliminary experiments were performed in order to verify the potential impact of the mycotoxin DON on the tubulin cytoskeleton. Incubation of A431 cells with DON (0.1, 1, 10 µM) for 24 h caused a concentration-dependent alteration of the appearance of the intensity of filaments of α-tubulin (Tubulin Fig. 1a, green). Image analysis of the fluorescence associated to the immunolocalization of tubulin confirmed the results with a significant decrease of the signal intensity starting from the incubation with 1 µM DON. Concomitantly, a concentration dependent increase of the average nuclear area was observed (Fig. 1b). Moreover, in order to give an insight into the potential cytotoxicity of DON in A431 cells, the average number of cells/optical field was manually quantified. This analysis revealed a tendency toward the increase in the number of cells incubated with 0.1 µM DON (Fig. 1c) and a significant decrease of the cell number in wells incubated with 10 µM DON (Fig. 1c).

### Effect of DON on tubulin and lysosome trafficking

**Figure 2:**
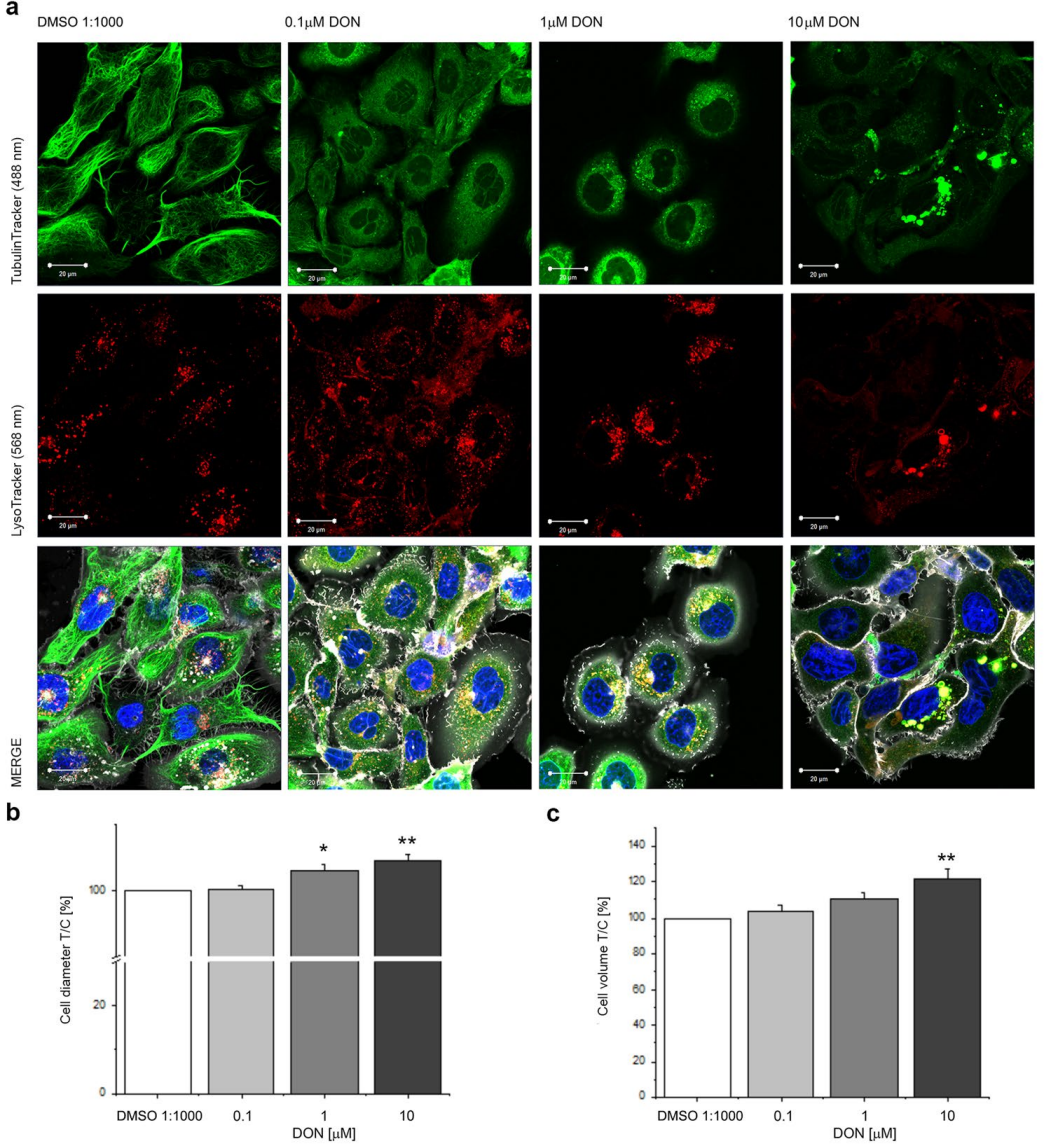
Effect of DON on living cells. (**a**) Appearance of A431 cells after incubation with DON showing tubulin (TubulinTracker, green), lysosomes (LysoTracker, red) and merged channels with plasma membrane (white) and nuclei (blue). Scale bars are equivalent to 20 µm. (**b**) Effect of the incubation with DON (grey bars) on cellular diameter. (**c**) Effect of the incubation with DON (grey bars) on cellular volume. Data are expressed as variation [%] in comparison to solvent controls (DMSO 1:1000; white bars) and presented as mean ± S.E. (n = 7). *Indicates at 0.05 level and **at 0.01 level significantly different distributions in comparison to cells incubated with 0.1 µM DON (Mann-Whitney test).

**Figure 3:**
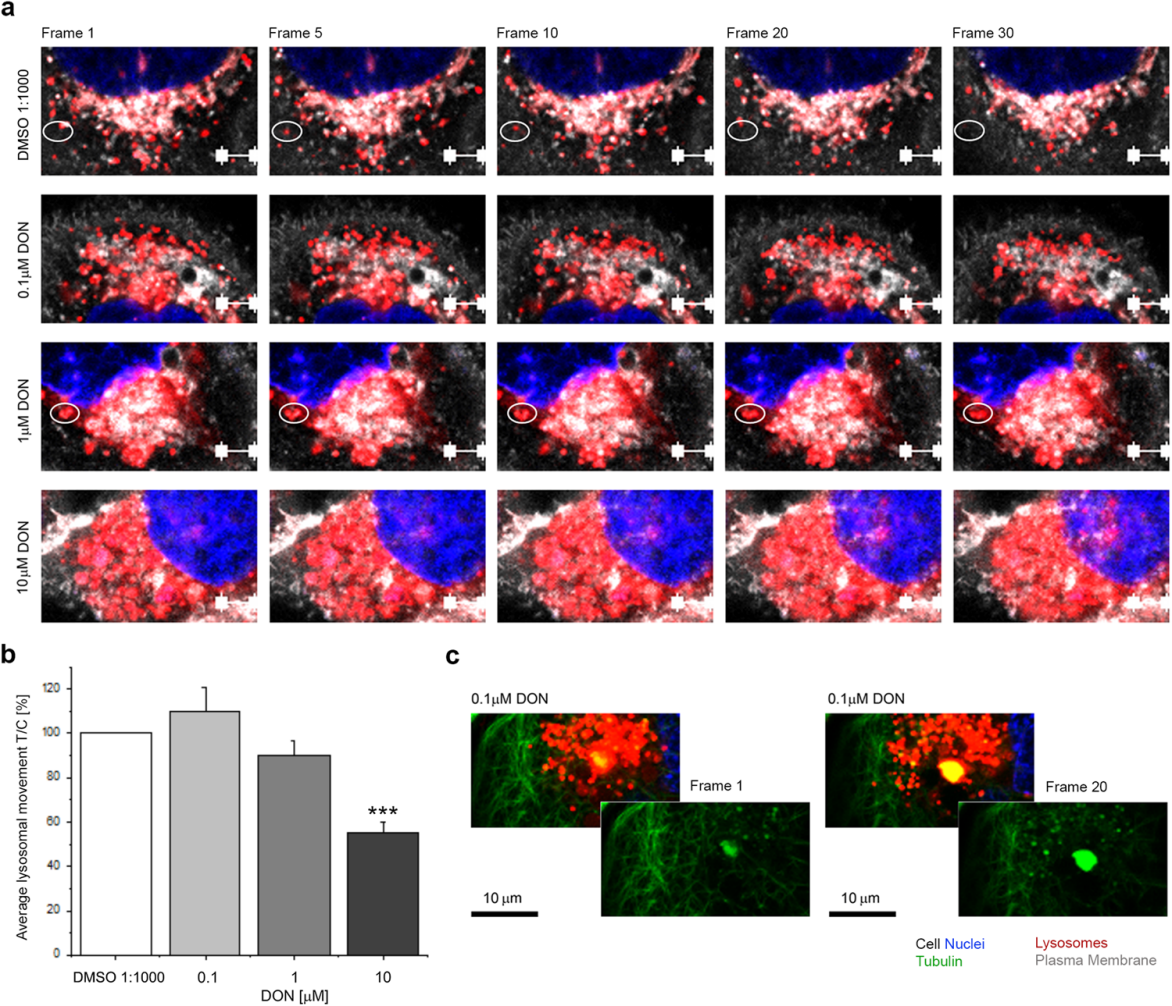
(**a**) Representative images acquired during the time series of the live cell localization of lysosomes in A431 cells. Lysosomes are depicted in red, cell nuclei in blue and plasma membrane in white. Scale bars (white) are equivalent to 5 µm. White circles highlight fixed position. (**b**) Quantification of lysosomes movement/trafficking. Data are expressed as mean ± S.E. of n = 5 independent movies analyzing for every experiment minimum 4 different cells. ***Indicates at 0.001 significant difference in comparison to No Effect Level (0.1 µM DON, ANOVA). (**c**) Representative interaction (yellow) between lysosomes (red) and tubulin (green) after incubation with 0.1 µM DON (24 h).

In order to verify if the alteration of the appearance of the tubulin cytoskeleton observed by immunofluorescence could also have a functional impact on living cells, live cell imaging experiments were conducted. Imaging was performed after 24 h incubation with DON (Fig. 2) and confirmed a concentration-dependent effect on the appearance of the tubulin network (Fig. 2a), associated with a progressive clustering of the lysosomes in the perinuclear region (Fig. 2a, in red). In parallel, automated evaluation of cell diameter and volume confirmed a progressive alteration of cell dimensions associated with toxin incubation (Fig. 2b,c). Moreover, in order to verify if the alteration of the tubulin cytoskeleton induced by the mycotoxin might have an impact on the movement/trafficking of lysosomes, sequential time series of the same optical field were also acquired. Representative frames, acquired at regular intervals are presented in Fig. 3a. Accordingly, the organelles and/or small lysosome clusters moved more rapidly in control conditions (DMSO 1:1000) and in cells incubated with DON (0.1 µM). Moreover, it was possible to observe areas of high motility/clustering of lysosomes corresponding to areas of aggregation of tubulin (Fig. 3c). Cells incubated with 1 and 10 µM DON were characterized by both, an accumulation in the perinuclear region of the organelles, and by progressive decrease of their movement (Figs 2a and 3a,b).

### Effect of DON on the response of A431 cells to uniaxial stretching: cell viability and morphology

**Figure 4:**
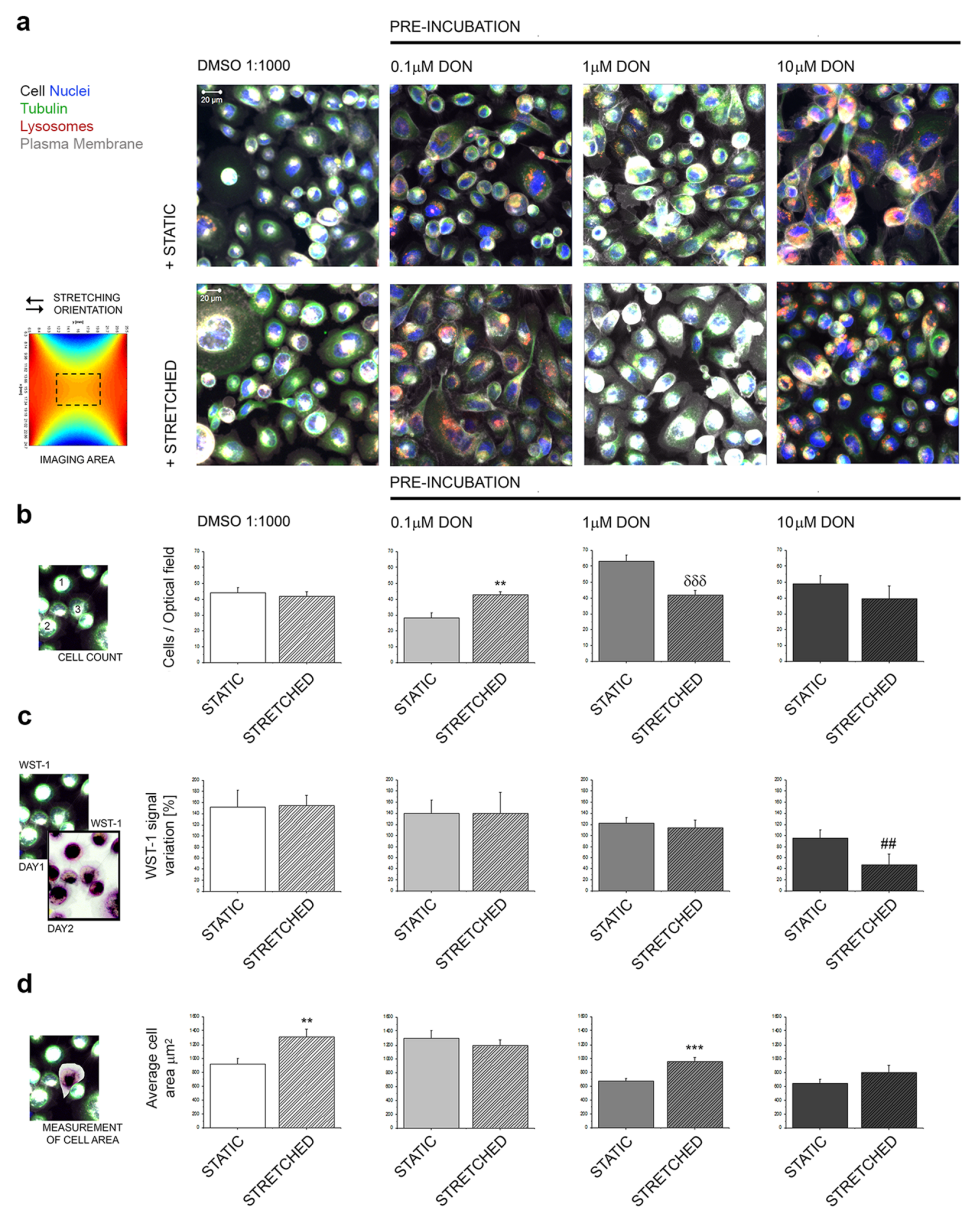
Effect of pre-exposure to DON on the capability of A431 to respond to mechanical stimulation. (**a**) Images are representative of A431 cells after incubation in static (+STATIC 24 h) or mechanically stimulated environment (+STRETCHED 24 h, 0.5 Hz, 15% deformation) showing cell nuclei (blue), lysosomes (red), tubulin (green) and plasma membrane (white) after live cell imaging. (**b**) Quantification of the average cell number/optical fields (o.f.); n ≥ 10 randomly acquired optical fields from at least 3 independent cell preparations. *Indicates at 0.05 level significant increase in comparison to STATIC incubation. ^δδδ^Indicates at 0.001 level significant decrease in comparison to STATIC incubation (Mann-Whitney test). (**c**) Measurement of cell viability. WST-1 data are expressed as % of cell growth between PRE-POST incubation (n ≥ 3 membranes for each experimental condition). ^##^Indicates at 0.01 significant decrease in comparison to STATIC and STRETCHED controls (DMSO 1:1000; ANOVA). (**d**) Average cell area; n ≥ 45 cells randomly chosen from 3 independent experiments (**and ***indicates at 0.01 and 0.001 level significant increase in comparison to STATIC incubation with Student’s *t*-test). Data are expressed as mean ± S.E. and describe controls (DMSO 1:1000, white graphs) and cells pre-incubated with DON (grey graphs) at static incubations (solid bars) and mechanically stimulated incubations (striped bars).

Considering the effect of DON to alter the tubulin cytoskeleton and associated physiological processes, we investigated the effects of the mycotoxin on the cellular capability to respond to uniaxial cell stretching. Control cells incubated under static and stretched conditions presented similar cell numbers and also similar proliferation rates (DMSO 1:1000; Fig. 4a–c), however an increase of the average cell area was observed in cells mechanically stimulated (Fig. 4a,d). 24 h pre-incubation with 0.1 µM DON did not cause any alteration in comparison to the behavior of the control cells, even if a moderate variation in the cell number was discernable (Fig. 4a,b). Pre-incubation with 1 µM DON seemed to alter the proportion between the number of cells/optical field and cellular area in A431 incubated in static conditions and mechanically stimulated (Fig. 4a,b,d). In both experimental conditions (i.e. STATIC and STRETCHED incubation) a progressive tendency toward the decrease of proliferation was noticeable (% proliferation 1 µM DON + STATIC: 121.67 ± 10.97%, 1 µM DON + STRETCHED: 113.78 ± 14.83%; controls STATIC 151.96 ± 30.39%; controls STRETCHED 154.05 ± 19.61%; Fig. 4c). In cells pre-incubated with the highest concentration of toxin the proliferation rate was significantly compromised in stretched cells (Fig. 4c; 10 μM DON) together with a more prominent effect on cellular morphology (Fig. 4a,d, 10 μM DON).

### Effect of DON on the response of A431 cells to uniaxial stretching: tubulin cytoskeleton

**Figure 5:**
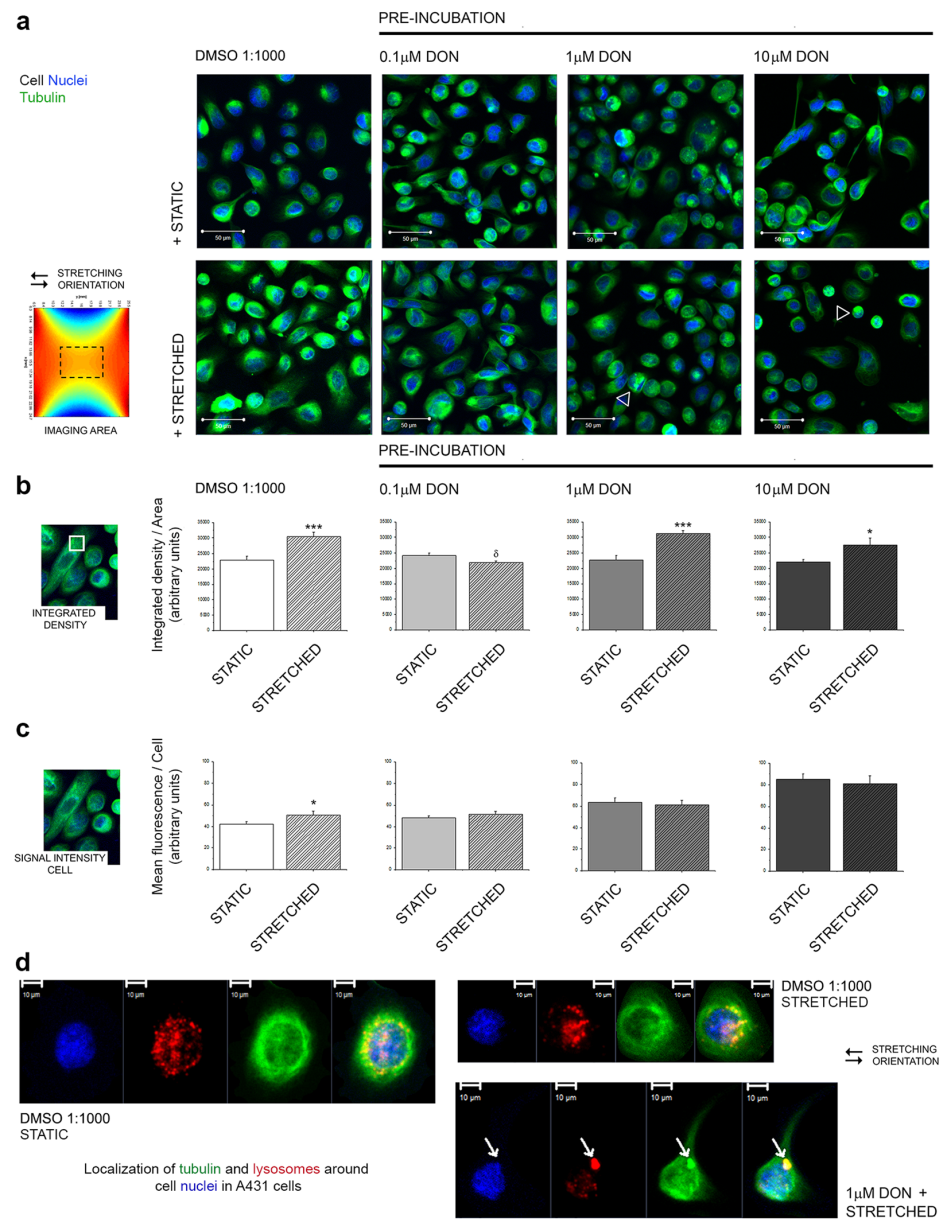
Effect of pre-exposure to DON on the tubulin cytoskeleton of A431 cells. (**a**) Images are representative of A431 cells after incubation in static (+STATIC 24 h) or mechanically stimulated environment (+STRETCHED 24 h, 0.5 Hz, 15% deformation). Cell nuclei (blue) and tubulin (green) after live cell imaging. White arrow-heads indicate cells with altered morphology and tubulin distribution. (**b**) Quantification of the integrated density of the TubulinTracker/area. (**c**) Mean fluorescence/cell. Data are expressed as mean ± S.E. and describe controls (DMSO 1:1000, white graphs) and cells pre-incubated with DON (grey graphs); static incubations (solid bars) and mechanically stimulated incubations (striped bars) and result from the quantification of n ≥ 45 cells randomly chosen from 3 independent experiments. *And ***indicate with Student’s *t*-test at 0.05 and 0.001 level significant increase in comparison to STATIC incubation. ^δ^Indicates at 0.05 level with Student’s *t*-test significant decrease in comparison to STATIC incubation. (**d**) Representative images of the distribution of the cellular lysosomes (red) with the tubulin (green) around the nucleus (blue): white arrows indicates co-localization (yellow) of TubulinTracker and LysoTracker in cells pre-incubated with 1 µM DON.

Since the pre-incubation with DON seemed to affect the capability of A431 cells to respond to the biomechanical stimulation, more focused analysis was performed on the tubulin cytoskeleton (Fig. 5a). In control conditions, cells mechanically stimulated for 24 h presented an increase of the tubulin signal, both, after quantification of the integrated density of the signal per area, and the quantification of the mean fluorescence/cell (Fig. 5b,c; DMSO 1:1000). In cells pre-incubated with 0.1 µM DON, the stretching protocol failed to trigger the increase of the signal of the cytoskeletal element (Fig. 5b,c). In cells pre-incubated with 1 or 10 µM DON, the application of the stretching protocol caused a clear morphological alteration of A431 cells (Fig. 5a) that was associated with an increase of the tubulin signal (Integrated density/area Fig. 5b; 1–10 µM DON). Several areas of co-localization of TubulinTracker and LysoTracker were observed (yellow Fig. 5d, 1 µM DON + STRETCHED), whereas control cells were generally characterized by a more even distribution of the cytoskeleton and the organelles (Fig. 5d DMSO 1:1000).

### Effect of DON on the response of A431 cells to uniaxial stretching: lysosomes

**Figure 6:**
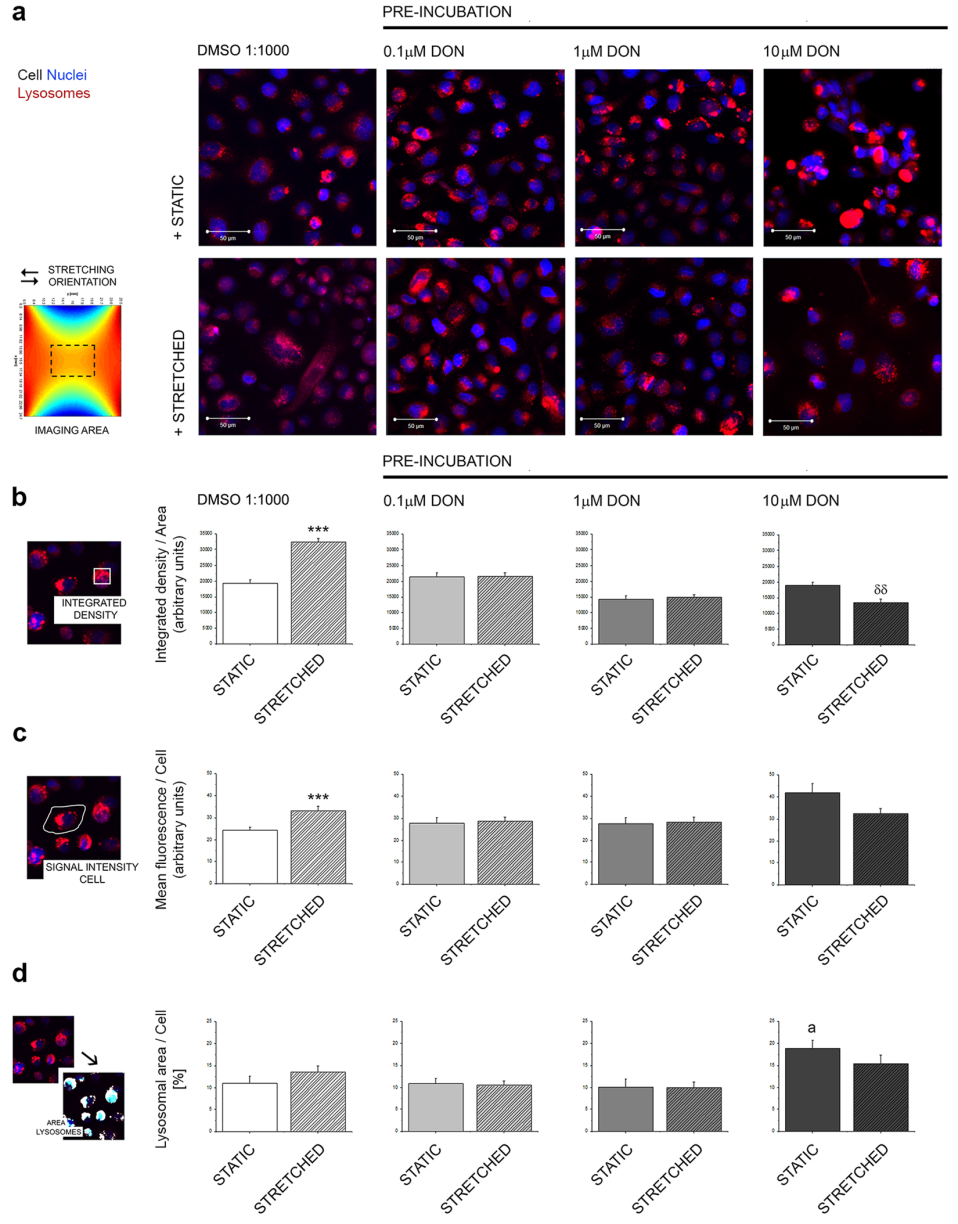
Effect of pre-exposure to DON on the lysosomes of A431 cells. (**a**) Images are representative of A431 cells after incubation in static (+STATIC 24 h) or mechanically stimulated environment (+STRETCHED 24 h, 0.5 Hz, 15% deformation) cell nuclei (blue) and lysosomes (red). (**b**) Quantification of the integrated density of the LysoTracker/area. (**c**) Mean fluorescence/cell. (**d**) % of cellular area covered by lysosomes. Data are expressed as mean ± S.E. and describe controls (DMSO 1:1000, white graphs) and cells pre-incubated with DON (grey graphs); static incubations (solid bars) and mechanically stimulated incubations (striped bars) and result from the quantification of n ≥ 45 cells randomly chosen from 3 independent experiments. ***Indicates with Student’s *t*-test at 0.001 level significant increase in comparison to STATIC incubation. ^δδ^Indicates with Student’s *t*-test at 0.01 level significant decrease in comparison to STATIC incubation. ^a^Indicates significant increase in comparison to STATIC CONTROLS with Student’s *t*-test at 0.05 level.

Incubation with DON proved to alter the properties of tubulin cytoskeleton upon the application of the uniaxial stretching and therefore parallel studies were performed to verify also the effects of the toxin on lysosomes (Fig. 6a). In control conditions, cells that underwent the mechanical stimulation protocol presented an evident increase of the intensity of the signal associated with the localization of the organelles (Fig. 6b,c; DMSO 1:1000). However, the percentage of the cellular area covered by the lysosomes remained constant (Fig. 6d; DMSO 1:1000). In cells pre-incubated with 0.1 and 1 µM DON this effect was abolished (Fig. 6b,c). On the contrary, cells that were pre-incubated with 10 µM DON, were characterized by high values of fluorescence intensity/cell, but also by an increase of total cellular area covered by the lysosomes, as suggested by the visual appearance of prominent red-colored areas in the optical field (Fig. 6a–d).

### Effect of biomechanical stimulation on the toxicity of DON

**Figure 7:**
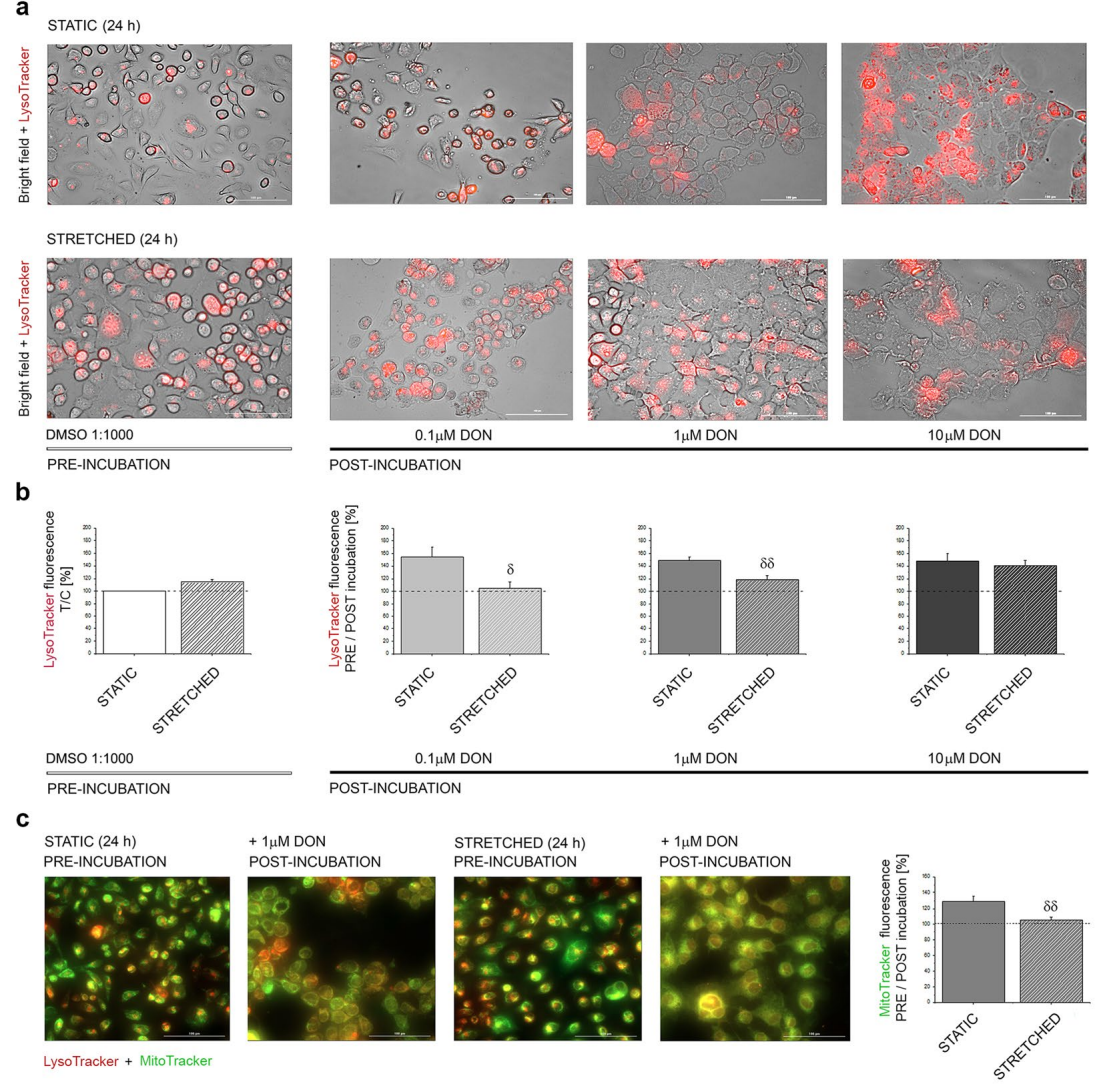
Impact of biomechanical stimulation on the toxicity of DON. (**a**) Merged images between bright field imaging and fluorescence imaging of the lysosomes (LysoTracker, red). (**b**) Quantification of LysoTracker fluorescence. Data describe controls (DMSO 1:1000, white graphs) and cells incubated with DON (grey graphs) depicting in solid bars static incubations and in striped bars the mechanically pre-conditioned cells. (**c**) Images of lysosomes (LysoTracker, red) and mitochondria (MitoTracker, green) and quantification of MitoTracker fluorescence. Data are average of the quantification of n ≥ 12 randomly acquired optical fields from at least 3 independent cell preparations. Data are expressed as mean ± S.E. For every treatment δ, δδ indicate at 0.05 and 0.01 level significant decrease of the signals in comparison to cells maintained in STATIC conditions (Mann-Whitney Test).

Exposure to DON significantly altered the physiological properties of A431 cells and hampered their capability to respond to uniaxial cyclic stretching. For this reason, it was decided to perform also experiments following a reversed protocol in order to verify if biomechanical preconditioning might modulate the capability of A431 to respond to the toxin. Initially, imaging and quantification of the fluorescence associated with the lysosomes were performed (Fig. 7a,b). In line with the previous data (Figs 5a and 6a), cells that underwent mechanical stimulation were characterized by a more elongated morphology associated with an increase of the signal of the LysoTracker. After incubation with DON, cells maintained in static conditions were characterized by a strong increase of the lysosomal signal. On the contrary, in cells pre-incubated with the stretching protocol the effect was reduced (Fig. 7b; 0.1 and 1 μM DON). However, the effect appeared to be concentration dependent and in cells incubated with 10 μM DON no difference was further detectable (Fig. 7b). Moreover, in order to obtain a preliminary insight if the effect of the stretching protocol followed by the incubation with DON could also have an impact on the physiological function of the lysosomes, an additional staining for the mitochondria was also included (MitoTracker; Fig. 7c). Mitochondria turnover is part of the normal physiological function of the lysosomes and selected experiments were performed labeling both organelles. In control conditions, the stretching protocol induced a slight decrease of the mitochondrial signal (−4.39 ± 3.05%) in association with an increase of the lysosomes (+14.76 ± 3.49%). Upon static incubation with 1 µM DON, an increase of the signal of the MitoTracker was observed, which appeared less pronounced in cells that were mechanically preconditioned (STATIC +28.83 ± 6.91%; STRETCHED +5.27 ± 3.89%; Fig. 7c).

### Impact of DON on the proteome of A431 cells

**Figure 8:**
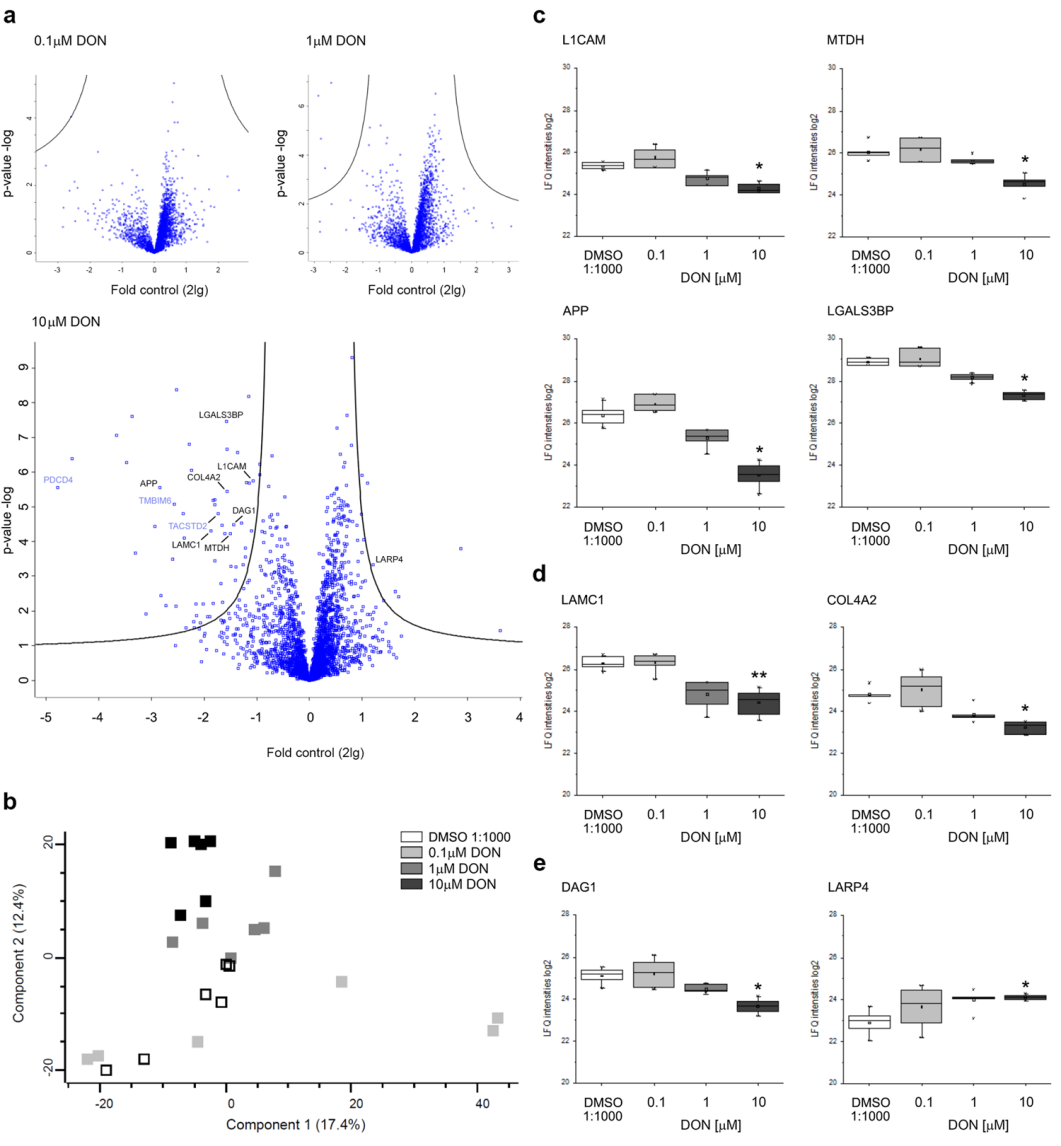
Effect of DON on the proteome profile of A431 cells. (**a**) Volcano plots highlighting DON induced proteome alterations compared to solvent controls (DMSO 1:1000). Fold control values are logarithmic on the base of 2 (X axes) p-values are logarithmic on the base of 10 (Y axes). Black borders delineate significantly regulated proteins with an FDR < 0.05. Proteins (gene names) associated with apoptosis regulation are depicted in light blue. (**b**) Clustering and separation of 10 µM and 1 µM DON treatment groups from 0.1 µM DON treatment and solvent controls (DMSO 1:1000) obtained with Principle Component Analysis (PCA). Representative protein alterations triggered by DON mediating cell adhesion (**c**), extracellular matrix composition (**d**) and cytoskeleton regulation (**e**) expressed as label-free abundance values. L1CAM (neural cell adhesion Molecule L1), MTDH (protein lyric), APP (amyloid-beta A4 protein), LGALS3BP (galectin-3-binding protein), LAMC1 (laminin subunit gamma-1), COL4A2 (collagen alpha-2 (IV) chain), DAG1 (dystroglycan) and LARP4 (La-related protein 4). *Indicates q values < 0.05; **indicates q values < 0.01 compared to control. Data are mean of n = 3 independent experiments analyzed in technical duplicates.

Since the toxic effect of DON appeared to be strongly interconnected with the capability of cells to respond to the biomechanical stimulation, untargeted proteomic analysis was performed in order to increase the understanding of the molecular effects triggered by the toxin in A431 cells. 24 h incubation with 0.1 µM DON showed no significant alterations of the proteome profile. Four proteins were found significantly downregulated upon treatment with 1 µM and 66 proteins were found deregulated upon treatment with 10 µM DON (Fig. 8a). Principal component analysis confirmed the concentration-dependency of the abovementioned effects, and the specificity of the response (Fig. 8b). Among the regulatory events, the incubation with 10 µM DON significantly downregulated several proteins governing cell adhesion like the neural cell adhesion molecule L1 (L1CAM), protein lyric (MTDH), the amyloid-beta A4 protein (APP) and the galectin-3-binding protein (LGALS3BP; Fig. 8c). Moreover, significant alterations were found also in proteins organizing the extracellular matrix (ECM), such as the laminin subunit gamma-1 (LAMC1) and collagen alpha-2 (IV) chain (COL4A2; Fig. 8d) and in proteins regulating cytoskeleton dynamics such as the dystroglycan (DAG1) and the La-related protein 4 (LARP4; Fig. 8e).

## Discussion

The capability to actively respond to the surrounding stimuli is one of the essential features of living creatures and can be extended from single cells to multicellular organisms. In this respect, it is easy to foresee the potential importance of the cross-talk between biomechanical stimulation and the development of cytotoxic processes. Biomechanical stimulation can have effects at several levels on cellular physiological functions, modulating, for instance, protein synthesis^50,51^, cytoskeleton dynamics^52^ as well as cell survival^8^. DON is one of the most prevalent mycotoxins worldwide^26^ and, in light of its relevance, it was chosen to explore the cross-talk between cytotoxicity and biomechanical stimulation. In more general terms, the performance of experiments in a mechanically stimulated environment and the comparison of cytotoxicity between static and dynamic environment is of crucial importance both for the definition of the impact of the mechanical stimulation in toxic processes and for the development of *in vitro* models that more efficiently could mimic *in vivo* conditions. Being trichothecenes well-known food contaminants, many efforts have been devoted to their study at intestinal level^53–55^. However, in addition, other target organs/tissues are emerging^46,47,56^. Epithelial cells are constantly subject to mechanical stimulation and A431 cells appeared to be an appropriate model for the elaboration of a test system that includes the investigation of the combinatory effect of uniaxial cell stretching and mycotoxins.

The first step toward the connection of the effect of DON and its potential to disrupt the physiological behavior of A431 cells was to describe the effects of the compound on the tubulin cytoskeleton (Fig. 1). DON triggered in A431 a distinctive alteration of the appearance of the tubulin network; this effect was in agreement with previous studies describing that the toxin can disrupt the actin cytoskeleton together with an alteration of connexin 43 and focal adhesion kinases ultimately leading to the impairment of cellular migratory capabilities^57^. Moreover, DON was reported to affect microtubule dynamics during the formation of the meiotic spindle^43,58^ having a direct impact on oocytes maturation. Similarly, incubation with DON triggered a functional impairment also in A431 cells. In fact, the toxin not only modulated cellular morphology, (i.e. cell size and diameter Fig. 2b,c), but also affected directly the functional capability of the tubulin network, as suggested by concentration-dependent impairment caused by DON on the lysosome trafficking (Fig. 3). The observed progressive clustering of lysosomes in the perinuclear region is a well-known behavior of these organelles^38^ and suggests an increase in the activation of the autophagic processes, pointing to an initial activation of this pathway as reaction to the effect of the mycotoxin. This response has been already described in several occasions^42,59^ and, in our experimental conditions it seemed to be directly related also to the aggregation of tubulin, (Figs 3c and 5d). These effects became detectable upon incubation with 0.1 µM DON, a concentration that can be considered not toxic in many cancer cell lines^60^. In agreement, incubation of A431 cells with 0.1 and 1 µM DON did not induce a significant decrease of the total protein content as measured with SRB assay (Supplementary Materials Fig. S1a); however, a significant decrease was observed in the protein content of the cytosolic faction, as showed in the Bradford quantification (1 µM DON; Supplementary Materials Fig. S1b). At sub-toxic concentrations, DON tended to moderately enhance proliferation of A431 cells (Fig. 1), as well as to have an impact on the balance between cell area/cell number in cells cultivated on the stretchable PDMS membrane (Fig. 4b and d). These data seem to sustain the concept that low concentrations of DON can exert pro-proliferative stimuli on tumor cells^60^, as well as on skin^47^.

In line with the characterization of the potential of DON to induce functional impairments in A431 cells, further experiments were performed to verify if the exposure to the toxin could affect the capability of the cells to respond to mechanical stimulation. Initially, it was decided to investigate the fate of the cells after the removal of the toxin. In this scenario, it would have been possible to appreciate both, the potential of biomechanical stimulation to influence the recovery of the cells in comparison to static incubation conditions and, additionally, to verify, if the exposure to an insult could decrease the capability of the cells to respond to the uniaxial cell stretching. It is well known that biomechanical stimulation can have a positive effect on cellular proliferation^61^ as well as to have a trophic effect on several elements of the cytoskeleton^62,63^. Incubation of A431 cells in the cell stretcher proved to be effective in increasing the intensity of the signal of tubulin cytoskeleton (Fig. 5b,c). These data are in agreement with previous studies that reported up-regulation of the gene expression of alpha tubulin in dermal fibroblasts after stimulation in similar conditions (16% deformation, 24 h cyclic stretching at 0.5 Hz)^45^. This response seemed to be abolished by pre-incubation with DON (0.1 µM), thus suggesting that even sub-toxic concentrations of the mycotoxin can have an impact on the cellular capability to respond to the uniaxial stretching. Paradoxically, in cells incubated with 1 and 10 µM DON an increase of the fluorescence signal of the TubulinTracker was observed. This event can be read in light of the huge morphological changes that A431 cells underwent when pre-exposed to the highest concentrations of the toxin. In fact, areas of accumulation of the TubulinTracker were visible in cells pre-incubated with 1 µM DON (Fig. 5a). In cells pre-incubated with 10 µM DON the effect was even more prominent due to a loss of characteristic cell morphology and increased cell rounding (Fig. 5a). In line with this interpretation, untargeted proteomic analysis revealed that DON modulated several proteins related to the cellular capability to respond to the surrounding environment. Incubation with the mycotoxin triggered a concentration-dependent downregulation of transmembrane proteins mediating cell adhesion (i.e. L1CAM, MTDH, APP and LGALS3BP; Fig. 8c)^64–67^, as well as a significant effect on components of extracellular matrix such as the laminin (LAMC1)^68^ and COL4A2 (Fig. 8d)^69^. In addition, dystroglycan was also downregulated (DAG1; Fig. 8e). The latter serves as bridge between the extracellular matrix and the cytoskeleton, playing a crucial role not only in ensuring cell adhesion, but also in transducing extracellular stimulation to the cells^70^. However, no variation was observed in the relative abundance of tubulin (Supplementary Material Fig. S3). These data seem to sustain the interpretation that the morphological alterations observed in the immunofluorescence experiments could be more likely the result of structural changes, increased turnover, post translational modification and/or the secondary effect of the downregulation of other proteins, rather than a direct effect of the toxin on tubulin (Fig. S3). Interestingly, upon incubation with 10 μM DON, LARP4 resulted significantly up-regulated. LARP4 is one of the important regulators of the cytoskeletal dynamics and it was previously demonstrated that its depletion could be associated to an alteration (increase) of cellular area^71^. In agreement, in our experimental conditions, evaluation of cell area after incubation with DON (Fig. 4d) revealed a concentration dependent decrease, even if the total cellular volume and diameter increased (Fig. 2b,c) thus suggesting a marked effect of DON on cell adhesion-spread; this could also account for the apparent slight differences between the cells cultivated on different substrates. In addition to the proteins regulating rather physical cell properties, another group of proteins involved in apoptosis regulation was found affected by the incubation with DON. This was evidenced by the downregulation of the anti-apoptotic protein bax inhibitor-1 (TMBIM6)^72^, the tumor suppressor PDCD4^73^ and the apoptotic gene regulator TACSTD2^74^ (Fig. 8a).

As a last step in the multiparametric workflow established for the evaluation of DON to alter the capability of A431 cells to respond to biomechanical stimulation, the intracellular distribution of lysosomes was also evaluated. In absence of DON (solvent controls, DMSO 1:1000) an increase of the signal of the organelles was observed after application of the stretching protocol (Figs 6b,c and 7a,b; DMSO 1:1000). This event could mirror a potential increase of autophagic processes in response to the stretching conditioning. In fact, an increase of autophagy in response to biomechanical stimulation has been previously reported^41^ and its importance in the maintenance of the physiological responses in relation to biomechanical stimulation has been recently reviewed^40,75^. Increase of the lysosomes upon uniaxial stretching, together with the trophic effects on tubulin, can be interpreted in light of a physiological improvement of A431 cells after biomechanical stimulation; the two elements combined seem to point toward the activation of controlled autophagic pathways, a well-known protective cellular mechanism^42,59,76,77^. Intriguingly, the quantification of the intensity of the LysoTracker/cell appeared to increase in both cells stimulated with the stretching protocol (DMSO 1:1000 Fig. 6a,c) and in cells exposed to the highest concentration of DON (10 µM, Fig. 6a,c). However, appearance of the signal was substantially different at the two conditions. In order to differentiate between an apparent physiological increase of the organelles and an imbalance, an additional image analysis workflow was applied for the evaluation of the relative cellular area covered by the organelles (Fig. 6d). In control conditions the percentage of cellular area covered by lysosomes maintained regular proportions, despite some variation of the intensity of the signals. This data in combination with the overall appearance of the cells suggests the increase triggered by stretching in control conditions (Fig. 6b,c) to be a physiological event. In addition, increase of the cellular areas upon biomechanical stimulation observed in control cells (Fig. 4d) decreased implicitly the likelihood that the alteration of the signal could be an optical artefact related to variation of cell morphology. This response was abolished by the pre-incubation with 0.1 and 1 µM DON, presumably sustained by the progressive alteration of cytoskeletal elements (Figs 5 and 8) and/or as a consequence of the structural cell remodeling (Fig. 2). The evaluation of cells pre-incubated with 10 µM DON demonstrated a significant increase of the cellular area covered by the organelles, suggesting a substantial alteration of lysosomes distribution, possibly related to the alteration of the dynamics of organelles movement (Fig. 3b) and cytoskeletal regulation also observed in the proteome analysis (Fig. 8e), as well as to cell morphological changes (Figs 2 and 4d).

For additional characterization of the cross-talk between biomechanical stimulation and the toxicity of the mycotoxin DON, further experiments were performed pre-incubating the cells in static or mechanically stimulated environment followed by the exposure to the toxin. Interestingly, the static exposure to DON triggered a concentration-dependent alteration of the appearance of the lysosomes and an increase of the signal of the organelles (expressed as percentage difference between pre- and post-incubation). On the contrary, in cells that were mechanically preconditioned, the increase was significantly lower (0.1 and 1 µM DON; Fig. 7b). This difference was no longer visible in cells that were incubated with the highest concentration of the toxin, suggesting that after a certain threshold the effect of the mycotoxin overcomes the effect of the training protocol. In order to give a preliminary insight into the pathophysiological meaning of these changes and potential repercussions on cell function, selected experiments were performed also applying a dye for mitochondria. In control conditions (DMSO 1:1000), the biomechanical stimulation caused a decrease of the mitochondrial signal, possibly related to intracellular redistribution and/or to a more efficient turnover of the organelles. Several studies previously demonstrated the importance of the autophagy-lysosome pathway for the maintenance of the correct turnover of mitochondria^78–81^ and this pathway was proven very important in the cellular resistance to trichothecene mycotoxins, like DON^82^. Interestingly, in cells incubated with 1 µM DON in static conditions it was possible to observe an increase of the signal of the MitoTracker in parallel to the increase of the LysoTracker (Fig. 7b,c). Recent studies related the adhesion protein L1 (significantly regulated also by DON; Fig. 8c) to mitochondrial trafficking, describing that the decrease of the protein could negatively regulate the motility of the organelles, as well as the fusion/fission equilibrium^83^. In line, the possible effects of DON on mitochondria could be extremely complex. In comparison to static incubation, cells mechanically preconditioned were characterized by a less pronounced increase of the lysosomal signal and even a more moderate increase of the mitochondrial signal (24 h incubation 1 µM DON; Fig. 7b,c). Even though the description of such complex mechanisms is far beyond the scope of the present paper, these data open new perspectives for the study of the possible relations between mitochondrial activation, lysosomes, biomechanical stimulation and DON.

In conclusion, the integration of biomechanical stimulation with cytotoxicity evaluation has proven to greatly enrich the evaluation of the effect of the trichothecene mycotoxin DON. In fact, DON impaired the capability of A431 cells to answer physiologically to uniaxial stretching and biomechanical stimulation modulated the effects of the toxin. Stretching of the extracellular matrix is normally not only well tolerated by cells, but may also improve their physiological function, and this data alone contributes to redefine the borders of toxicity of the mycotoxin. Being these effects detectable at concentrations of DON that in many assays are considered non-toxic, these data are of crucial importance for the proper *in vitro* evaluation of the mycotoxin.

## Materials and Methods

### Cell culture

For the characterization of the toxicity of DON in the biomechanical stimulated environment, the epidermoid carcinoma cell line A431 was used. Cells were cultivated in Minimum Essential Medium (MEM) supplemented with L-glutamine (4.5 g/L), 10% (*v/v*) heat inactivated fetal bovine serum (FBS) and 1% (*v/v*) penicillin/streptomycin and maintained in humidified incubators at 37 °C and 5% CO_2_. For the performance of the biomechanical stimulation experiments, cells were seeded on a polydimethylsiloxane (PDMS) membrane functionalized with Matrigel Matrix (Corning, Discovery Labware, MA; USA). At the day of the experiments, medium was substituted by phenol-red free medium D-2909 supplemented with 10% (*v/v*) heat inactivated fetal serum (FBS), 1% (*v/v*) penicillin/streptomycin and 25 mM HEPES (pH 7.4). If not otherwise specified, cell culture media and supplements were purchased from GIBCO Invitrogen (Karlsruhe, Germany), Sigma-Aldrich Chemie GmbH (Munich, Germany) and Sarstedt AG & Co (Nuembrecht, Germany), VWR International GmbH (Vienna, Austria) and Thermo Fisher Scientific GmbH (Vienna, Austria).

### Deoxynivalenol

DON was purchased from Romer Labs Diagnostic GmbH (Tulln, Austria). For cellular experiments, solid substance was dissolved in dimethyl sulfoxide (DMSO; Carl Roth GmbH, Karlsruhe, Germany) at the respective stock solutions were stored at −80 °C and diluted in cell culture medium (1:1000) for the performance of the experiments.

### Immunocytochemistry

For immunofluorescence analysis cells were seeded in 8-well microscopy slides (Falcon, Corning, New York, USA). After 48 h cells were incubated with DON (final concentration range 0.1–10 µM) or solvent control (DMSO 1:1000) for 24 h. Cells were rinsed with PBS (37 °C) and fixed with pre-warmed formaldehyde (3.7% in PBS, 15 min). Cells were then permeabilized with triton-X (0.2% in PBS, 10 min), washed and blocked with heat inactivated goat serum (10% in PBS, 1 h, RT). For the detection of tubulin the anti α-tubulin antibody (Tubulin; B-7; 1:1000 dil. Santa Cruz Biotechnology, Heidelberg, Germany) was used. Afterwards, fluorescent labeled anti mouse secondary antibody was added (1.5 h, Alexa Fluor 488 Donkey Anti-Mouse (A-21202) dilution 1:1500, Life Technologies, Thermo Fisher Scientific, Waltham, USA). After removal of secondary antibody slides were post-fixed with 3.7% formaldehyde (10 min, RT); at the end of the post-fixation 100 mM glycine was used to mask reactive sites and slides were mounted with Roti-Mount FluorCare DAPI (Carl Roth GmbH, Karlsruhe, Germany) and sealed. Images were acquired with a Confocal LSM Zeiss 710 equipped with ELYRA PS. 1 system using a Plan Apochromat 63×/1.4 oil objective (zoom 1.5) and an Andor iXon 897 (EMCCD) camera.

### Live cell imaging

For the evaluation of cellular responses to both incubation with DON and uniaxial stretching, live cell imaging experiments were performed. Cellular structures/organelles were stained with specific dyes: cell membrane was stained with CellMask™ Deep Red Plasma membrane Stain (1:1000 dilution, white), lysosomes with LysoTracker® Red DND-99 (1:1000 dilution, red, indicated as LysoTracker), mitochondria with MitoTracker® Green FM (1:1000 dilution, green, indicated as MitoTracker), tubulin with TubulinTracker™ Green (1:2000 dilution, green, indicated as TubulinTracker), and cell nuclei with Hoechst 33258 (1:1000 dilution, blue). Staining solutions were diluted in Live Cell Imaging Solution (all from Molecular Probes, Life Technologies, Thermo Fisher Scientific, Waltham, USA). At the end of the staining, cells were rinsed and maintained in Live Cell Imaging Solution for the microscopy analysis. Time series and confocal images were acquired with Confocal LSM Zeiss 710 equipped with ELYRA PS. 1 using a Plan Apochromat 63X/1.4 oil objective (Figs 1–3), whereas membranes were imaged with a Plan Neofluar 10X/0.3 (zoom 4; Figs 4–6). Moreover, membranes were also analyzed with the Cytation3 Imaging Multi-Mode Reader (BioTek, Winooski, VT, USA) for image acquisition and evaluation of the fluorescent signal (objects-mean per optical field; Fig. 7).

### Automated evaluation of cell diameter and cell volume

For the determination of the cell size and cell diameter a CASY TT Cell Counter and Analyzer (OMNI Life Science GmbH & Co. KG, Bremen, Germany) was used. To this aim, the range of dimensions measured was set constant for every experiment and used also for the regular cell counting, with the cursor position optimized for the cell line of interest (minimum diameter detected 10.6 μm). At the end of the incubation with the toxin, cells were gently detached from the petri dishes with accutase and re-suspended in medium to a final volume of 1 mL. Mean volume and diameter of the cells were evaluated injecting in the instrument 100 µL of the cell suspension diluted in the measuring solution CASY Ton, as specified by the supplier. Data are expressed as µm (Diameter) and fL (Volume). Data are presented as average of the quantification of 13 different samples obtained from 7 independent cell preparations (biological replicates).

### Biomechanical Stimulation

In order to verify the influence of biomechanical stimulations on the effect of DON, different experiments were performed with the help of the cell stretcher^44^. This device is able to deliver accurate, repeatable and controlled uniaxial strain cycles to a PDMS membrane on which a population of cells can be cultivated. During the experiment, the cells are stimulated mechanically by experiencing cyclic deformation. The upper limits for the frequency of stimulation and the amplitude of the strain are 10 Hz and 20%, respectively. In the context of the experiments, 15% strain mechanical stimulation was applied for 24 h at a frequency of 0.5 Hz. For the evaluation of the impact of DON on the capability of A431 cells to respond to biomechanical stimulation (Figs 4–6) cells were pre-incubated for 24 h with DON and afterwards maintained in static or biomechanically stimulated environment in toxin free medium (schematic workflow Supplementary Material Fig. S2). Biomechanical stimulation experiments were always carried out in parallel and the results compare directly static and stretched incubation conditions (Figs 4–7). Since the biomechanical stimulation was differentially distributed on the PDMS membrane, the methods of choice for the evaluation of the biological effects of the stretching in combination with exposure to DON were live cell imaging coupled with image analysis and fluorescence signal quantification. In fact, these methods allowed the evaluation of the area of the membrane that was more consistently stimulated by the stretching protocol. To this aim a multi-parametric workflow was established including image analysis with several endpoints (average cell number, cell area, integrated density of the fluorescence signal/area, mean intensity of the fluorescence/cell and for lysosomes also % of the cell area occupied by the organelles), moreover information about cytotoxicity were provided with a non-destructive method (WST-1 assay).

### Image Analysis

Analysis of the raw data of the images was performed with software ZEN 2012 SP3 (black; Carl Zeiss, Jena, Germany). Analysis of lysosomes movement after staining with LysoTracker was performed with the Tracking plugin of Fiji free software. Data are the results of the quantification of n = 5 independent movies, considering for each movie 4 different cells and following at least 5 trajectories (particles) per cell. For evaluation of the (*i*) average cell area (*ii*) mean fluorescence/cell (*iii*) area covered by lysosomes/cell all images were processed consistently prior to quantification and in order to avoid bias, the channels of interest were temporarily disabled during the selection of the cells within the optical fields. In parallel, integrated density of the fluorescence signal/area was performed independently with the free software ImageJ as previously reported^84,85^. In order to avoid the possibility that *ad interim* evaluation could influence the selection/quantification process, statistical evaluation of the datasets obtained with ImageJ and ZEN 2012 SP3 were performed only at the end of all the analyses. Each data point is the average of the quantification of minimum 45 cells/region acquired from 3 independent experiments, thus resulting from the random selection, for each experiment, of 5 cells in 3 different optical fields (o.f.). Moreover, since the mechanical stimulation is not uniform on the membranes, image acquisition was performed consistently in the central region of the PDMS substrates, as schematically depicted in the relevant figures. In order to rule out the possibility that the differences in the quantification between static and stretched cells could be due to focus variations during the stimulations protocol, the signal of the nuclei was also quantified (Supplementary Material S4). In this case, no major differences were observed between static and stretched cells.

For both, image analysis with ZEN 2012 SP3 (black) and with ImageJ, respective background correction was applied. For the images acquired with the Cytation3 instrument, fluorescence intensity was expressed as object (cell) mean per optical field. For every experimental condition data are mean of at least 12 randomly acquired optical fields from 3 independent cell preparations.

### Assessment of cytotoxicity

In order to give an indication about the cellular proliferation or cytotoxic potential of the experiments performed with the cell stretcher, WST-1 assays were performed in selected steps of the experimental layout. Briefly, Cell Proliferation Reagent WST-1 (Roche Diagnostics GmbH, Mannheim, Germany) was diluted 1:10 (v/v) in phenol-red free DMEM-2909 and applied to A431 cells for 45 minutes. Subsequently, supernatant was transferred in a 96 well plate and absorbance was measured at 450 nm (reference wavelength 650 nm) with a Victor^3^V 1420 Multilabel Counter Plate Reader (Perkin Elmer, Waltham, USA). Afterwards, cells were rinsed with PBS and, according to the experimental layout, further incubated in static or stretched conditions or stained for live cell imaging. For each experimental condition, measurements of the supernatants were performed in triplicate and data (solvent controls; DMSO 1:1000, 0.1, 1, 10 µM DON) are average of 3–5 independent experiments.

Sulforhodamine B (SRB) experiments presented in Supplementary Fig. S1 (Supplementary Materials Fig. S1a) were performed according to the protocol of Skehan and co-workers^86^. Briefly, at the end of the incubation, cells were rinsed with pre-warmed PBS, fixed with 50% trichloroacetic acid (30 min, 4 °C) and stained with SRB reagent (0.4% in 1% acetic acid, 1 h, RT). Stained cells were rinsed with diluted acetic acid (1%) and bi-distilled water. In the end, protein-bound SRB reagent was diluted in Tris base (10 mM) and absorbance was read at 570 nm with a Cytation3 Imaging Multi-Mode Reader (BioTek, Winooski, VT, USA).

### Proteomic analysis

For the proteomic analysis, cells were seeded in 6-well plates, allowed to settle for 48 h and incubated with DON (0.1, 1, 10 µM) or solvent control (DMSO 1:1000) for 24 h. At the end of the incubation, samples were processed using a classical bottom up proteome analysis strategy; to this aim high-resolution mass spectrometry was employed as described in detail previously^87^. In short, cells were lysed, protein content in the cytoplasmic fraction was determined using a Bradford assay (Bio-Rad Laboratories, Vienna, Austria; Fig. S1b), and sample aliquots were digested with Trypsin/Lys-C mixture (Promega Corporation, Madison, WI, USA). Peptides were separated by nano-flow UHPLC (Ultimate 3000RSLC Thermo Fisher Scientific, Austria) and analyzed with a Q Exactive orbitrap mass spectrometer (Thermo). Data analysis was accomplished using the MaxQuant 1.6.0.1 software^88^ including the Andromeda search engine and the UniProt database for human proteins (version 102014 with 20,195 entries). For protein identification, a FDR < 0.01 was applied both on peptide and protein level with at least two peptides identified per protein. For label free quantification, a FDR < 0.05 was applied to a two-sided T-test and a minimum of a twofold abundance difference.

### Statistical Analysis

Data were analyzed with the help of the software OriginPro 2016G (OriginLab Corporation, Northampton, USA). Multiple comparison of independent samples was performed with one way ANOVA test followed by Fisher test for pairwise comparisons. Mann-Whitney and Student’s *t*-tests were applied for the direct comparison of groups of data. Distributions were considered different using threshold values of 0.05. Proteome data were analyzed with the Perseus statistical analysis package^89^ An overall false discovery rate (FDR) smaller than 0.05 was applied for significant regulatory events. q-values corresponding to corrected p-values for multi-parameter analysis were obtained for each protein.

### Data Availability

The mass spectrometry proteomics data have been deposited to the ProteomeXchange Consortium via the PRIDE^90^ partner repository with the dataset identifier PXD008996 and are available at http://www.proteomexchange.org/. Additional representative images acquired during independent biological experiments (corresponding to Figs 4–6) are available in Supplementary Materials Fig. S5. Further information about the datasets generated during and/or analyzed during the study are available on request from the corresponding author (giorgia.del.favero@univie.ac.at). Questions/requests concerning the cell stretcher can be addressed directly to O S (sbaizero@units.it).

## Electronic supplementary material

Supplementary material
