# Supplementary material for: Deoxynivalenol induces structural alterations in epidermoid carcinoma cells A431 and impairs the response to biomechanical stimulation
Source: Sci Rep. 2018 Jul 27;8:11351. doi: 10.1038/s41598-018-29728-5 (PMC6063857; doi:10.1038/s41598-018-29728-5)
Supplement: Supplementary file 1 — Supplementary material [file 41598_2018_29728_MOESM1_ESM.pdf]

SUPPLEMENTARY MATERIALS

Deoxynivalenol induces structural alterations in epidermoid carcinoma cells A431 and impairs the response to biomechanical stimulation

Giorgia Del Favero[1*], Lydia Woelflingseder[1], Lukas Janker[2], Benjamin Neuditschko[2], Stefano Seriani[3,4], Paolo Gallina[3], Orfeo Sbaizero[3], Christopher Gerner[2], Doris Marko[1]

1 Department of Food Chemistry and Toxicology, Faculty of Chemistry, University of Vienna. Währingerstr. 38-40, 1090 Vienna, Austria

2 2 Department of Analytical Chemistry, Faculty of Chemistry, University of Vienna. Währingerstr. 38-40, 1090 Vienna, Austria

3 Department of Engineering and Architecture, University of Trieste Via A. Valerio 10, 34127 Trieste, Italy

4 Robotik und Mechatronik Zentrum, Deutsches Zentrum für Luft- und Raumfahrt e.V. (DLR), Oberpfaffenhofen, Germany

* Corresponding author. Dr. Giorgia Del Favero (giorgia.del.favero@univie.ac.at)

Department of Food Chemistry and Toxicology, Faculty of Chemistry, University of Vienna. Währingerstr. 38-40, 1090 Vienna, Austria.

Fig. S1

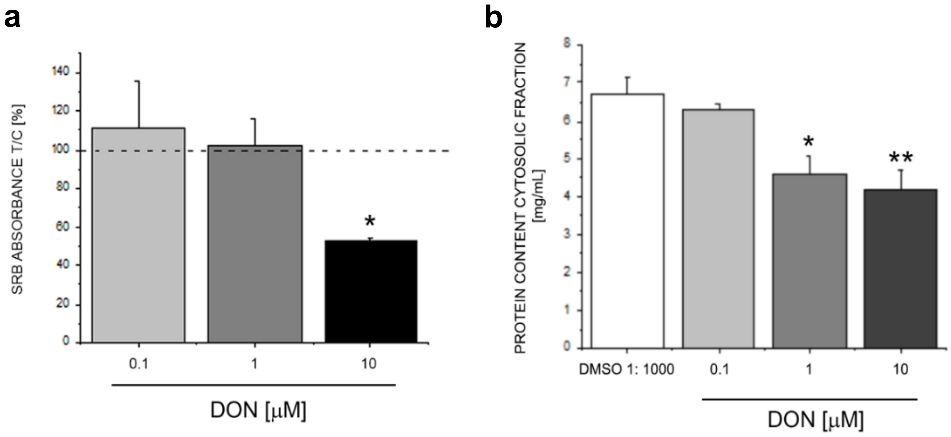

Supplementary Materials Fig. S1. Protein quantification in A431 cells after 24h incubation with DON. (**a**) SRB assay. Data are expressed as test / control [%] and depicted as mean ± S.E. n=3-4 independent experiments performed in triplicate. (**b**) Bradford assay quantification of protein content in the cytosolic fraction. */** indicates at 0.05 and 0.01 level different population means in comparison to incubation with 0.1 µM DON (**a**) or solvent control (DMSO 1:1000; **b**) ANOVA.

Fig. S2

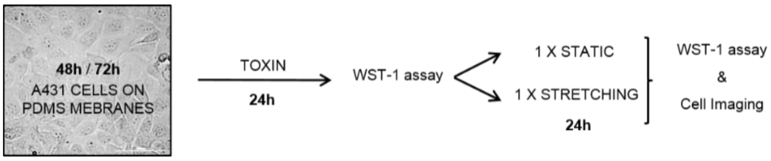

Supplementary Materials Fig. S2. Schematic representation of the experimental workflow of the results presented in Figures 4; 5; 6.

Fig. S3

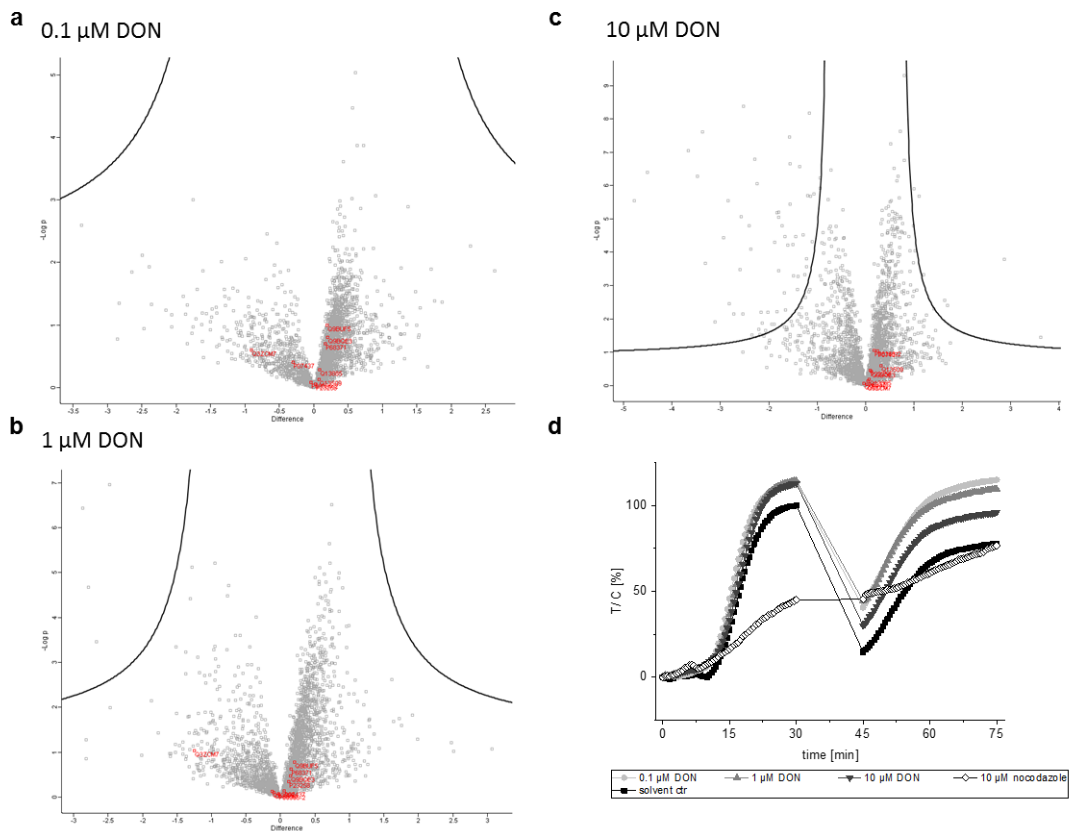

Supplementary Materials Fig. S3. Volcano plots describing DON-induced proteome alterations in A431 cells. In red tubulin isoforms (Q9BQE3; P68366; P07437; Q13885; Q13509; P68371; Q9BUF5; Q3ZCM7; P23258) expressed by A431 cells and relative abundance after incubation with DON 0.1 µM (**a**), 1 µM (**b**) and 10 µM (**c**). Effect of DON (0.1-10 µM) on tubulin polymerization (**d**) in comparison to solvent control (black) and tubulin polymerization inhibitor Nocodazole (10 µM, white). Tubulin polymerization assay was performed with a commercial kit (17-10194 *In vitro* Tubulin Polymerization Assay Kit (≥ 99% Pure Bovine Tubulin) from Chemicon, Merck Millipore according to the specification of the supplier.

Fig. S4

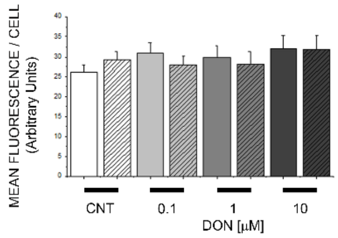

Supplementary Materials Fig. S4. Mean fluorescence signal of cellular nuclei stained with Hoechst 33258. Solid bars refer to static incubation and striped bars refer to stretched incubation of control cells (white background) and pre-incubation with DON (grey background). Data are expressed as mean ± S.E. and quantification is the result of n ≥ 45 cells randomly from 3 independent experiments.

Fig. S5

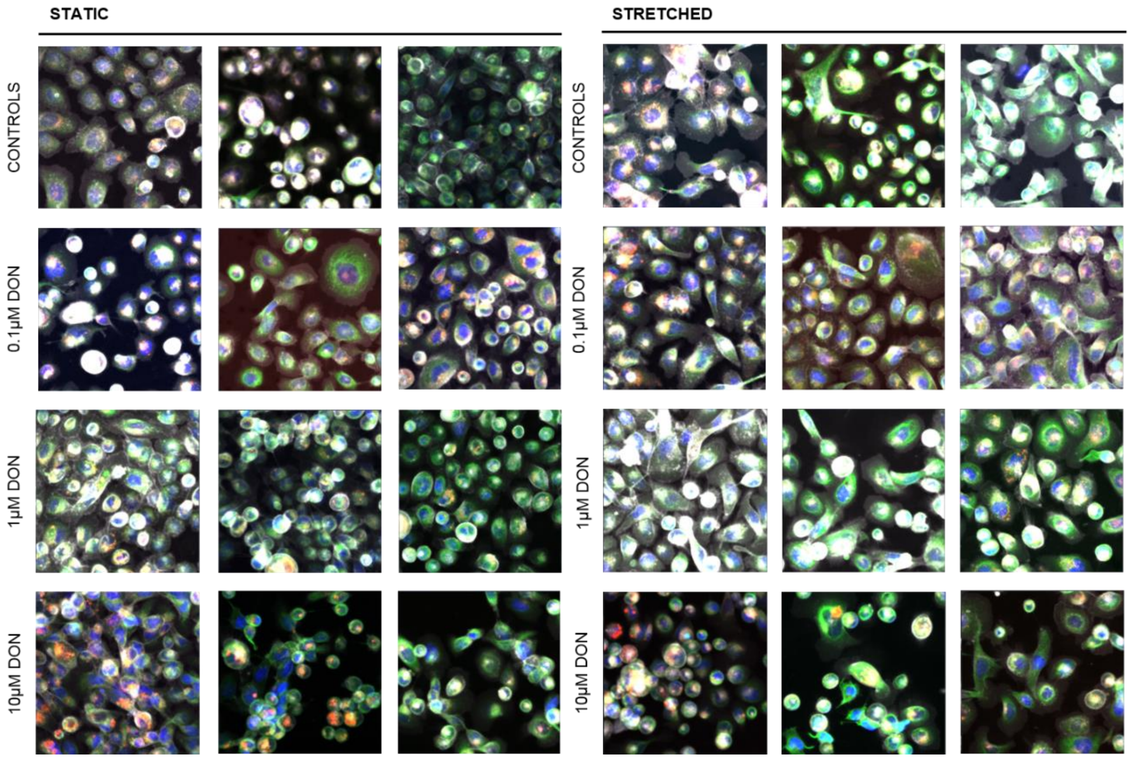

Supplementary Materials Fig. S5. Additional representative images acquired during the performance of the experiments. Static and Stretched membranes were always incubated in parallel and each column has been obtained with a different cell preparation.
